# Stakeholder Views on Active Cascade Screening for Familial Hypercholesterolemia

**DOI:** 10.3390/healthcare6030108

**Published:** 2018-08-31

**Authors:** Carla G. van El, Valentina Baccolini, Peter Piko, Martina C. Cornel

**Affiliations:** 1 Department of Clinical Genetics and APH Research Institute, Amsterdam UMC, Vrije Universiteit Amsterdam, 1081 GT Amsterdam, The Netherlands; mc.cornel@vumc.nl; 2 Department of Public Health and Infectious Diseases, Sapienza University of Rome, 00185 Rome, Italy; valentina.baccolini@uniroma1.it; 3 MTA-DE Public Health Research Group of the Hungarian Academy of Sciences, Faculty of Public Health, University of Debrecen, 4028 Debrecen, Hungary; piko.peter@sph.unideb.hu; 4 Department of Preventive Medicine, Faculty of Public Health, University of Debrecen, 4028 Debrecen, Hungary

**Keywords:** familial hypercholesterolemia, cascade screening, sociotechnical analysis, stakeholder analysis, personalized prevention

## Abstract

In familial hypercholesterolemia (FH), carriers profit from presymptomatic diagnosis and early treatment. Due to the autosomal dominant pattern of inheritance, first degree relatives of patients are at 50% risk. A program to identify healthy relatives at risk of premature cardiovascular problems, funded by the Netherlands government until 2014, raised questions on privacy and autonomy in view of the chosen active approach of family members. Several countries are building cascade screening programs inspired by Dutch experience, but meanwhile, the Netherlands’ screening program itself is in transition. Insight in stakeholders’ views on approaching family members is lacking. Literature and policy documents were studied, and stakeholders were interviewed on pros and cons of actively approaching healthy relatives. Sociotechnical analysis explored new roles and responsibilities, with uptake, privacy, autonomy, psychological burden, resources, and awareness as relevant themes. Stakeholders agree on the importance of early diagnosis and informing the family. Dutch healthcare typically focuses on cure, rather than prevention. Barriers to cascade screening are paying an own financial contribution, limited resources for informing relatives, and privacy regulation. To benefit from predictive, personalized, and preventive medicine, the roles and responsibilities of stakeholders in genetic testing as a preventive strategy, and informing family members, need to be carefully realigned.

## 1. Introduction

Familial hypercholesterolemia (FH) is one of the most common human inherited disorders, characterized by high levels of serum total cholesterol and low-density lipoprotein cholesterol (LDL-C). Two forms are characterized according to the mode of inheritance of the disease (autosomal recessive and autosomal dominant FH). Both forms lead to premature atherosclerosis and cardiovascular problems [1,2]. The autosomal recessive form is an extremely rare condition worldwide (except in the island of Sardinia, Italy), and it is caused by defects in the *LDLRAP1* gene [3]. With prevalence ranging between 1:200 and 1:500 worldwide, autosomal dominant FH is the most common form which is caused by gene mutations, most notably in the low-density lipoprotein receptor (*LDLR*) gene, or defects in genes involved in cholesterol metabolism *(APOB*, *PCSK9*). In this paper, we will focus on the autosomal dominant type of FH. The diagnosis of FH is based on high concentrations of LDL-C, family history of hypercholesterolemia, presence of premature coronary artery disease, and cholesterol deposition in the form of xanthomas and/or arcus senilis [4]. Genetic testing can confirm a diagnosis in about 80% of cases, and international guidelines differ on the importance of genetic testing for treatment [5,6]. Currently, FH is globally underdiagnosed and undertreated [1]. The risk of cardiovascular events can be reduced by medication via statins, and adopting a healthy lifestyle [7]. The early diagnosis and treatment initiation is critical for patients with FH. The risk of premature death from cardiovascular disease is four times greater for untreated FH patients than it is for those who do not suffer from FH [8]. Due to the autosomal dominant inheritance of this type of FH, first degree family members have a 50% chance of also carrying the same pathogenic gene variant. Genetic screening of family members allows for personalized prevention, and it is paramount to find and inform these family members in a timely manner.

An important question is how family members—who, at that point, are still considered to be healthy and feel healthy, but are nevertheless at risk—can be traced and informed in an ethically responsible and efficient manner? Newson and Humphries [8] differentiated direct and indirect strategies. In a direct approach, healthcare professionals or representatives from a screening program contact family members directly, after having obtained the addresses from the proband or index patient in whom the mutation has been detected. Another indirect option is that the proband or index patient contacts his or her family members. Newson and Humphries argue that direct contacting is efficient and ethically justifiable in certain conditions. For instance, the index could inform the family members first that he or she has had genetic testing, and that the family will be contacted soon with more information. In this way, receiving an unsolicited letter form a healthcare provider might be less of a shock. As family dynamics may differ, how best to approach the family should be discussed with the proband, who might take up a more or a less active role [9].

In several European countries, initiatives were started to identify FH patients and trace family members using various approaches. The Netherlands is among the very few countries where a systematic screening program was introduced, and proved an inspiration to other countries [10,11]. The Dutch screening started in 1994 [12], was developed into a national screening program in 2004, and was characterized by the proactive tracing, direct contacting, informing, and testing of healthy family members of an index patient. After the addresses were obtained from the index, patient family members were asked to be visited and tested at home by a nurse employed as a genetic field worker at the Foundation for Tracing Familial Hypercholesterolemia (StOEH). For the index, patient complex genetic testing was performed at the laboratory of the Amsterdam University Medical Center. Once the mutation was identified, family members were only tested for the familial mutation, which was relatively cheap. The pedigrees and mutations were registered in a database, and whenever a new index was identified, the database was checked to see if this person might be related to a known family in the database, so genetic testing could be personalized. The program was found to be cost-effective [13]. The programme had always been regarded as a project, and when it started, it was expected that most carriers would have been detected by 2010, based upon an estimated heterozygote frequency of 1:500. When evidence accrued that the heterozygote frequency was actually >1:250, it became clear that not all carriers could be found by that year. The funding was extended from 2010 to 2013. Between 2004 and 2014, 15,000 FH patients were identified [8]. During this decade, financial support was provided by the Ministry of Health, and the program was coordinated by the National Institute of Public Health and the Environment (RIVM). Though successful, the screening program ended in 2014, leaving an estimated 40,000 of the expected 70,000 mutation carriers in the Netherlands unidentified. After 2014, other actors in regular healthcare were supposed to take over, coordinated by the newly established foundation LEEFH (Dutch Expertise Centre for Inheritance Testing of Cardiovascular Diseases). The budget was reduced, and strategies to approach healthy relatives had to be reconsidered. In the regular healthcare system, official inhabitants of the Netherlands have to purchase obligatory insurance of a “basic healthcare package”, for which persons with a low income receive governmental reimbursement. The insurance covers the financial costs after falling ill, and includes “indicated prevention” for persons at an individually increased risk, but not population screening and vaccination programs. The latter are typically funded from public health budgets. For individual healthcare cost, an “own risk” of 385€ (in 2018) applies for medication and hospital expenses per year, including laboratory testing. Since the ending of the publicly funded screening program in 2014, people may have to pay up to the amount of the own risk to get tested.

After ending the screening program, the yearly number of index patients identified almost halved, to about 141 in 2015. However, most notably, the number of family members identified dropped when family screenings dwindled from over 2000 to around 400 in 2015 [14,15].

Healthcare professionals were concerned and pleaded to continue the screening program, also drawing attention to the fact that less children with FH would now be identified [16]. In 2016, the Dutch Minister of Health stated that the proactive and direct approach, as it had been conducted at the time of the screening program, would not be reinstalled [17]. Though the ending of the Dutch screening was not unanticipated, a new argument surfaced. Patients should make autonomous choices in making use of healthcare or screening, and home visits were seen as too “paternalistic”. In other words: the acceptability of the screening program was questioned by policymakers. FH care had to be integrated in regular healthcare, while for the tracing of family members, the government pointed to existing facilities in clinical genetics. Following standards in clinical genetics, it was stressed that the index patient is responsible for informing family members, who can then decide whether they want to make an appointment for discussing genetic testing [14,17]. Healthcare professionals can support the index by, for instance, giving family letters to the index, so he or she can distribute these to family members.

The history of the Dutch screening program and its recent abandoning of a proactive and direct approach provides us with an excellent opportunity to explore the pros and cons of various approaches to informing healthy family members on their genome. In an era of increasing possibilities for predictive, preventive, and personalized medicine, the question how to balance these pros and cons is topical.

This study explores opinions on how active healthcare professionals can or should inform (healthy) family members when a patient is diagnosed with a hereditary condition, using FH as an example. By identifying and interviewing a range of relevant stakeholders, we aim to clarify arguments for and against more proactive, direct approaches, and show varying responses to and requirements for informing family members, given a changing landscape with new regulatory restraints. For the stakeholders involved, this can contribute to a process of mutual learning and attunement of their practices. By making these arguments and choices available to an international audience, we hope to add to the international discussion on best practices in tracing and informing healthy family members on their genome.

## 2. Materials and Methods

Literature on cascade screening for FH in the Netherlands and national policy concerning the program was studied, stakeholder groups were selected, and interviews with six persons from five stakeholder groups were conducted.

The stakeholders were identified based on a conceptual model for a sociotechnical analysis. In a sociotechnical analysis the roles and arguments of different actors or stakeholders in a network are studied to understand how an innovation, such as genetic testing, becomes part of, and shapes, a new healthcare practice [18,19]. In this process, also, the conditions for use and capacities of a technology become more clearly defined (co-evolution of technology and social-ethical context). In this model, the sources of dynamics may stem from various domains, requiring stakeholders in other domains to adapt their practices. Technological innovation in the scientific domain, for instance, may challenge current practice. New findings or changes, in demand by public and patients, may require healthcare professionals to adapt the organization of services. Also, evaluation of acceptability of procedures by regulatory, advisory, and governmental agencies may provoke changes in healthcare. In our analysis, we will use this conceptual model to describe how stakeholders in The Netherlands are realigning their practices in genetic testing and informing family members in a changing healthcare landscape, after the ending of the FH screening program in 2014. In this case, changes in technology were not the source of dynamics.

After the screening program ended, the Minister argued that the field itself, meaning the stakeholders in FH care, had the responsibility to organize effective care themselves. We will therefore focus our attention to stakeholders in the healthcare and patient domain of the model, to see how they are responding to changed viewpoints in the policy domain on the acceptability of an active approach contacting family members directly, and are trying to reorganize their practices.

**Figure 1:**
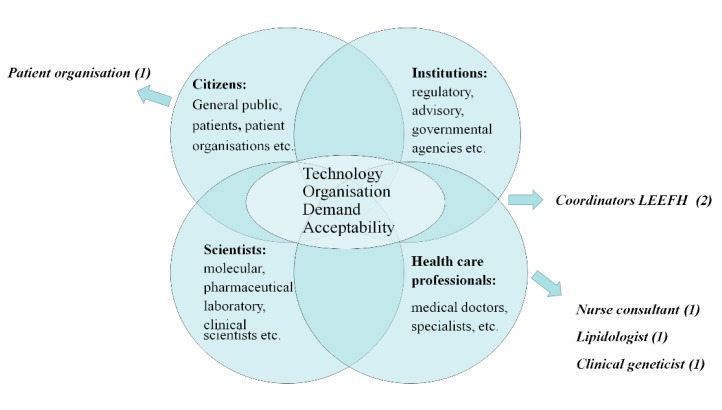
Network of stakeholders that need to be attuned in transition processes that could be initiated by dynamics in technology, organization, demand, and/or acceptability in healthcare systems (adapted from Rigter et al., 2014, [19] and Achterbergh et al., 2007, [18]).

Based on the model in Figure 1, we identified five relevant stakeholders, and conducted five interviews in total with six persons (see Table 1): an official from the patient organization, two professionals from the official Netherlands’ expertise center for familial cardiovascular disorders that coordinates the detection and care of FH patients (LEEFH), a lipidologist, a clinical geneticist, and an FH nurse consultant that informs patients and advises them on informing their family members.

The stakeholders came from three regions in the Netherlands, and included staff members of national organizations (LEEFH and patient organization) that were expected to be knowledgeable on screening policies, as well as healthcare professionals from local services in hospitals in these three regions. The clinical geneticist and lipidologist were at the senior management level and well known with local and national FH policy. The stakeholders were not representing their organizations, but were asked to reflect on informing family members given their professional experiences. A topic guide was constructed, asking about the pros and cons of different approaches to informing family members in the old screening program, the current situation, and the ideal situation, both in practical and ethical terms (see Supplementary Material S1: interview protocol stakeholder views). The interviews were semi-structured, and were held face-to-face. For LEEFH, two staff members with different functions were present at the interview, and they are listed as Coordinator 1 and 2. All authors were present during at least one interview, one author was present at all interviews (C.G.v.E.).

Respondents were informed about the research aims, and asked for consent prior to the interviews to audio record the interview, store the transcript, and use quotes. Since the consent did not include full disclosure of the transcripts, these are not provided as Supplementary Material. After the audio-recorded interviews were transcribed verbatim, the transcripts were sent to the interviewees for a member check. Thematic analysis was performed manually by two persons (C.G.v.E. and M.C.C.). The themes were inspired by the conceptual framework and established via inductive coding. The codes for two interviews were discussed until consensus was reached. The other interviews were coded based on this list by C.G.v.E. The code list is available as Supplementary Material (Table S1: Code list transcripts interviews “stakeholder views on active cascade screening for familial hypercholesterolemia 2018”).

The project proposal was sent to the Ethical Review Board of VUMC, that decided that the Dutch Medical Research Involving Human Subjects Act (WMO) does not apply to the abovementioned study, and that an official approval of this study is therefore not required.

## 3. Results

When discussing the pros and cons of different approaches to informing family members, several themes became apparent: uptake, psychological burden, privacy, and autonomy (Section 3.1). In discussing the current situation or contemplating a future approach, roles and responsibilities were discussed (Section 3.2), while in addition, further needs were identified, such as raising awareness, and availability of resources (Section 3.3).

### 3.1. Pros and Cons of an Active Approach

The active approach of the former screening program was characterized by both a direct approach to contact family members after the index had given permission to do so, and the planning of visits at home or at the workplace to inform the family members, measure cholesterol and draw blood. Respondents noted the high uptake as an important benefit of the approach of the screening program.

*“We only asked them “who are your brothers and sisters?” and if it was okay, we contacted them and sent them letters… “Ok, in your family there is FH, do you want explanation?” and then within two weeks, we made a phone call to the brother asking “Did you receive this information? Is it clear? This is how the program works” and most of the time in 90% we made an appointment.”*

(Coord.:2)

*“[Now] we can only phone, and also the nurses at the expertise centers can only phone the index patients asking them “have you talked to your brother, children?” to stimulate them. Then we send all the packages [for testing, C.G.v.E.], but then we do not get them back. If you actively approach, which is what we did, that was very, very effective”.*

(Coord.:1)

In addition, the support for the index patient in informing family members was seen as an important advantage of the active approach. The lack of such support and the psychological burden for the index was seen an important drawback of the new system.

*“It’s also emotionally very difficult for the index patient to have to tell to his brother or sister who lives far away that he has a genetic disease and it’s wise to get checked and also that there are consequences for his or her children.”*

(Coord.:1)

Policy documents mentioned paternalism as a drawback of the previous proactive and direct approach, stressing that patients should be responsible to seek healthcare autonomously. However, respondents held various opinions whether this objection was important enough to ban such direct approaches, given the otherwise substantial health gain. In weighing pros and cons, ideas of patient autonomy play an important role, such as in policy, though the ways forward envisaged by the interviewed stakeholders, based on such notions, varied.

*“…the more autonomy the better it is, but to have autonomy you should be informed… The best thing would be a screening program again but even if we have it back, even then, more attention would be needed for information for consenting autonomous people. We always need it, whatever system we have.”*

(Patient Org.)

*“I think everybody is working 24 h a day, how good it is that you don’t have to come to an office to get your blood drawn from 9 to 10? No, I can understand it (that the old system was seen as paternalistic C.G.v.E.)…but my vision is completely different. I think it’s very modern and I think it would be good to continue that way because then you really get your samples and you find the patients.”*

(Coord.:1)

Privacy was seen as potentially at odds with direct contacting of family members, while also trespassing the right not to know was mentioned. As one respondent remarked:

*“It is not a reason to cut the program but this problem has always existed…very rarely someone informed me because (they said) “I’d rather not know”. The opposite happens many times.”*

(Patient Org.)

Respondents had various ideas on modernizing the active approach. These included directly approaching family members via email addresses obtained from the index to invite the family to contact healthcare.

*“I think it’s more of this time to first get this consent that you can approach these family members. I think by now most people have access to e-mail etc. So I think if we could have approached the family members directly, I would say by now you would do it by e-mail. And you will have a good e-mail about it and then you would ask the index patient: is it okay if I email this and this family member? And the index patient would say: you will receive an e-mail and they will inform you about this (FH) and if you don’t want it let me know. It’s true in a way that it’s a bit old fashioned and not very time effective to travel in a car and visit family members.”*

(Lipid.)

### 3.2. Roles and Responsibilities

With the termination of the screening program in 2014, stakeholders had to redefine their roles and responsibilities, given that the Minister had put the responsibility to organize effective care in their hands. In the following, we map views on the roles and responsibilities of the various stakeholders involved.

It took a while before a new coordinating organization was in operation, LEEFH. With very limited funding, they had to find and stimulate best practices. Whereas StOEH had been a centralized organization, LEEFH set up centers in various parts of the country where FH nurse consultants were installed. The way these consultants operate and the amount of time they have may differ per city, which may affect the level of support index patients have, and consequently, the uptake of cascade testing by family members.

*“So if they see that a LEEFH center is not producing enough family members they should say what is wrong how can we help or what is needed?”* (Lipid.). To perform the coordinating role is difficult, as one respondent put it there is *“lack of people, lack of knowledge, lack of education”*.

(Clin. Genet.)

What remained unchanged after the end of the screening program was that patients are treated at departments of internal or vascular medicine and/or in primary care. During the screening program after genetic testing, genetic field workers of StOEH discussed tracing family members with the patient. For the treating physicians, this was perceived as a *“black hole”* (Lipid.). For those in the new system, there is an advantage in now being able to focus on the family in the care pathway:

*“The disadvantage of the STOEH-system was that only once the pedigree was made; they didn’t have any follow-up. What we do now is every time the patient is here, we check and update the pedigree, maybe new children are born or people died.”*

(Lipid.)

The central role of the treating physician is also underscored by the respondents from LEEFH, and this role is compared to the involvement of clinical genetics. Respondents value clinical genetics for its experience with informing the family, which might be helpful to train other professionals, or coordinate screening. However, most notably, the cost and the extra referral for the patient are seen as a drawback.

*“Yes, we are trying also to collaborate and to learn from the expertise of the clinical geneticists: although it is not a very heavy disease and it’s very easily treated, the cascade screening is difficult. It is difficult to reach the family of the index patient and we think that clinical geneticists have much more experience with that but what we are trying to do is to keep the medical care of the index patients and their families where they belong, which is the internist or a cardiologist. That’s why we are making this whole network: we don’t want the patient to be referred to all kinds of different stations for finding his whole family. We want to give the advice at the local out-patient clinics so that’s what we are trying to do, to optimize that.”*

(Coord.:1)

The Minister had suggested to include clinical geneticists in FH care, as they are able to support the index patient because of their expertise in dealing with hereditary disorders. It was argued that FH could follow the model of cancer care, where the clinical geneticist would be able to directly approach family members in case this would be too burdensome for the patient [14,17]. While, in principle, clinical geneticists might directly approach family members, it is not clear if they actually do so, and how often, as the current Dutch guideline suggests that such a direct approach would only be advisable in exceptional circumstances, when the index patient is unable or unwilling to contact the family, and it is very important for the relative’s health [20].

*“We had a discussion…, that also in clinical genetics there is the discussion on what is the approach to the family members, what is allowed or not. In two ways, it is allowed on ethical grounds; are you allowed as a physician to contact a family member who doesn’t know anything, and also financial, because the healthcare insurance companies say “you can only act when there is a disease and the patients come to you”. But…an active approach…is prevention, it is not healthcare.”*

(Coord.:2)

Among clinical geneticists in the Netherlands, there is currently a difference of opinion, as especially in the north of the Netherlands, clinical geneticists support the idea of referring FH patients to clinical genetics. They argue cost can be contained, as the casemix will become more favorable by including more simple diagnoses, such as in the part of FH cases [21].

Respondents indicated to see the nurse consultants as ideally positioned to support the index patient, informing the family members:

*“But I think that the role of the genetic fieldworkers or nurses…always [have] more time to talk with people and to inform them than doctors; the specialist or also general physicians, they don’t take or have time, but nurse practitioners who do have some time…I think they should be more in charge in this process.”*

(Patient Org.)

Such nurses could be trained by clinical genetics and be connected either to the genetics department or internal medicine.

*“I think there is some plus in having nurse practitioners coming from the area in which the disease is involved. But you can also have nurse practitioners from the genetics. It can be possible. And probably a mix would be perfect. But they should be trained.”*

(Clin. Genet.)

The role of the general practitioner (GP) has become more important after the screening program ended. Not only does the GP have to be aware of FH, but also discussing the risk for family members and stimulating the patient to contact these relatives has become important, as there is no organization to do so. Respondents expressed concern that GPs did not have enough knowledge, and mentioned cases of GPs not referring for genetic testing. However, experiences of collaboration differ according to region:

*“So a lot of times the GP says do it at your place [hospital] but if a patient for some reason, e.g., economical reasons, says it’s too expensive in the hospital and I want to go back to my GP, we urge the GP to also check the family members.”*

(Lipid.)

The RIVM (National Institute of Public Health and the Environment) might be given a task to set up a central organization of cascade screening for all sorts of hereditary disorders.

*“I think this cascade screening is really aiming at…living better and longer. For all these [hereditary] diseases. So in my view this is prevention. And it should be paid not from the budget of health care but by the national prevention budget. And in my view here there is a task for RIVM … it should be a national program, a screening program for genetic known diseases in which there are actionable interventions. Then we have a national program, we have a national database and it is much cheaper and, more importantly, we can also give care at a very high systematic similar level to all these people. Because one of the problems now is that carriers are informed by the GP, by this person, by that person, all kinds of persons and they tell them very different stories… If we have a centre in which all care is coordinated and information is coordinated…[They] can be factually informed and really helped during their path.”*

(Clin. Genet.)

### 3.3 Resources and Awareness

When the cascade screening was an official program supported by the government, funding for the human resources and the DNA testing was provided from public health budgets. Now that regular healthcare has to integrate this preventive service, staff has to become available to actively support the provision of information to relatives. For counselling by internal medicine only recently has a tariff been agreed. Healthcare providers, such as lipidologists, or the nurse consultants in their department, are paid for counselling patients on informing their family members if their hospital also includes a genetics center. Clearly this raises a threshold for LEEFH centers operating in peripheral hospitals without a clinical genetics department.

A barrier for a direct approach is the fact that healthcare insurance only covers when an individual patient visits the doctor with a question. Organizing a prevention program is not covered:

*“[Health care insurance companies] have to agree that if we actively approach family members who might have FH that they are still insured although it’s the health care system that approaches them instead of them…approaching the health care system with a question.”*

(Lipid.)

*“Do they [health insurance companies] think that actively approaching FH patients or family members is insured healthcare or not. They’ve been too vague about it. So for me it’s unsure what they mean.”*

(Lipid.)

There is reimbursement to the individual patient for “indicated prevention” which would be requested by individuals at increased risk [22]. However, the cost of the program would not be reimbursed, unlike national screening programs.

Furthermore, the DNA test is covered by basic health insurance in the Netherlands, but the first 385€ (in 2018) of any hospital care or medication has to be paid by the individual patient, the so-called “own risk”. Thus, unlike national screening programs, the own risk is a barrier for healthy individuals considering genetic testing. Hospital care is, in the Dutch situation in general, not an offer to healthy persons, but driven by questions of diseased patients. Therefore, to allow for complete reimbursement of preventive services, the healthy relative of FH patients has to be fitted into the system of “insured care”.

Respondents indicated that after ending the program, an effort has to be made to make people and stakeholders, such as GPs, aware of hereditary forms of high cholesterol.

*“And an important difference between having a population screening and not having is awareness, because of…the capacity of publishing things, so people know it exists and especially general practitioners are remembered time to time of these people and there are happening things around such a program.”*

(Patient Org.)

*“You should have some way to reach big groups and that would be via media, television, radio, YouTube, Facebook. Because if people read things like that, then they start thinking maybe I should get tested…”*

(Consult.)

## 4. Discussion

In this study, we explored stakeholder opinions on how active healthcare professionals can or should inform (healthy) family members when a patient is diagnosed with FH. When discussing the pros and cons of different approaches to informing family members, several themes became apparent: uptake, psychological burden, privacy, and autonomy. Interviewees strongly valued the high effectiveness of the previously adopted active approach and direct contacting. In the new indirect approach, the autonomy of family members is underscored, as relatives should seek healthcare, while privacy of family members is protected as they are only approached by the index. Respondents thought patients should make autonomous choices, but to do so, good information and awareness is needed. The new system offers little support for index patients informing their family members, and the psychological burden can weigh heavily on the index, further reducing uptake. Resources for giving information and supporting the index in the new system are reduced, while tasks are relegated to regular healthcare, and stakeholders are still finding ways to develop best practices and define their roles and responsibilities in FH care, given new regulatory restraints.

Barriers to cascade testing mentioned by the stakeholders are paying an own financial contribution, limited resources for informing relatives, and privacy regulation. LEEFH is well-positioned to discuss best practices with the range of stakeholders in the field. Discussions with Dutch healthcare insurance have recently led to dedicated funds for nurse consultants being able to counsel patients and help them inform their family [15]. If this were extended to nurses working in hospitals without clinical genetic centers, informing relatives will become more sustainable. The fact that healthy persons have to pay for a preventive service (because of the “own risk”) is not yet solved. Well educated people with sufficient income might ask for FH-testing more often than people with low income and low health literacy. Thus, the current approach focusing on “own responsibility” might be less effective than the previous program, and might also increase inequity.

As for the privacy regulation, an important issue today is the different interpretation of the new General Data Protection Regulation (GDPR) in different hospitals, potentially making stakeholders reluctant to be more active. Clarity is needed on what is allowed and what not. If this is clear, more knowledge sharing among stakeholders on best practices on active approaches without direct contacting will assist in limiting this barrier. A promising practice mentioned by stakeholders was actively stimulating patients within current restraints, such as checking by phone if they succeeded in informing family members, and asking about informing the family during follow-up care.

Such options for a more active approach gained prominence during a public discussion that ensued after a Dutch BRCA family went to court [23]. In this family, a young woman got breast cancer which was found to be hereditary. The family was shocked to hear a pedigree had been made years before for another family member, but information about the mutation had not reached their branch of the family. This initiated discussion among healthcare professionals questioning what they are allowed to do in the era of new privacy laws. There is renewed discussion on the question of what the public expects of healthcare when information is available that is of relevance to them: is there a duty of care or a duty to inform? Geneticists had actually followed current guidelines, so officially, they were not to blame. However, the guidelines allow for support of the index patient, and geneticists themselves argued that they should be more active in overseeing the process of informing family members and check whether the index has been able to inform family members [24]. An initiative has started to develop new interdisciplinary guidelines on informing family members that can change the regulatory landscape and can also affect FH care. 

Challenges for the future, when more predictive, personalized, and preventive possibilities arise, include mainstreaming genetics. FH care can be an example, using the strengths of clinical genetics, in training other healthcare professionals on drawing pedigrees, counselling, emotional support, support in tracing and informing family members. With the increasing numbers of older citizens in EU countries, prevention is becoming a more central theme in healthcare. Especially in conditions where genetics offers a substantial and quantifiable risk estimate, and prevention is available, these preventive services should be prioritized.

More government involvement is needed as a formally organized screening program could standardize support and information, and lead to more equitable healthcare. Many countries, such as Poland, Norway, and the UK, now develop FH care with this new vision of genetic preventive medicine. We hope that the role of stakeholders elsewhere will be described, and support the further development of predictive, personalized, and preventive services.

## Figures and Tables

**Table 1:** Stakeholders interviewed.

Stakeholder	Roles in FH Screening	Abbreviation Interviews
Patient organization	Patient advocacy	(Patient Org.)
Coordinator LEEFH	Coordinating role in FH care; update database	(Coord.:1); (Coord.:2)
Nurse consultant	Inform about FH, make pedigree, support index with informing family by providing information	(Consult.)
Lipidologist	Internal medicine or vascular specialist, involved in FH diagnosis, care and treatment	(Lipid.)
Clinical geneticist	Genetic testing, counselling, support informing family members	(Clin. Genet.)

## Supplementary Materials

The following are available online at http://www.mdpi.com/2227-9032/6/3/108/s1, Table S1: code list transcripts interviews “stakeholder views on active cascade screening for familial hypercholesterolemia 2018”, Supplementary Material S1: interview protocol stakeholder views.
