# Supplementary material for: Stakeholder Views on Active Cascade Screening for Familial Hypercholesterolemia
Source: Healthcare (Basel). 2018 Aug 31;6(3):108. doi: 10.3390/healthcare6030108 (PMC6163326; doi:10.3390/healthcare6030108)
Supplement: Supplementary file 1 [file healthcare-06-00108-s001.pdf]

Table S1
Code list Transcripts interviews 'Stakeholder views on active cascade screening for familial hypercholesterolemia 2018'

reason screening		treatment/prevention	
end of screening programme		liberal ideas,	stakeholders organise themselves
		cost	
		should have gone on	
		not of these days	
active approach	con	paternalistic	
		right not to know	
		few complaints about transgressing right not to know/positive reactions	
		autonomy to inform the family	
		burden (not) informing family members	
	pro	support for patient informing family members	
		effective	
		cost effectiveness	
new system	LEEFH	LEEFH centers characterised by regional differences, own budgets, own means and eagerness	
		reduction in finding patients or family members	
		lack of awareness, public, GPs	
	role GP	few patients per practice, lack of knowledge,	
		unwilling to refer	
		lack of knowledge, wrong advice	
		make GP aware	
	nurse	should do the cascade screening	

	RIVM	regular care is not fit for FH care
		reports, letter sent by patient organisation, discussion Medisch Contact
	clinical genetics	FH is relatively simple genetic disease, easy treatment, no complex knowledge or counseling
		clinical genetics can coordinate/support/train cascade testing, without doing it itself
		costly
		Clinical genetics is focused on diagnosis not on saving lives through prevention
		new guidelines will be made
		family meetings can be organised, clinical geneticist can invite
	government	it is a task of the government that people know about FH
		RIVM should organise national cascade screening programme for hereditary diseases as FH
	awareness	Should stimulate awareness (via information campaign)
		autonomy: patient should decide if he wants to inform family members
privacy/legal issues		cannot inform family members without consent index, confidentiality
		treating physician should inform patient
		direct contacting may be problematic in light of privacy regulation
		failing to inform family members is also problematic, people can reproach
	future	also in other screening programmes people are selected based on risk
active without home visits		more information to make autonomous decision
		nurse/genetic field worker
		family gatherings/family consultations
		folder, websites
		stress that family testing can be done at GP so no own risk involved
		family meeting
StOEH		centralized, no regional connections
insured care		patient has to come with a question, you cannot invite people to screen
		own risk is a barrier
		separate organisation health care and prevention is problem

case finding	not all mutations known<br>pop up for physician ordering (cholesterol) testing
testing children	cord blood<br>test at age 7-8<br>importance of genetic testing for children
insurance	no (life) insurance problem if FH is treated
stakeholders	(NHG organisation of) GPs<br><br>(NIV Professional organisation) Internal medicine<br>cardiologists<br>pediatricians<br>professional societies clinical genetics<br>media television, facebook etc<br>Ministry of Health<br>ZIN (National Health Care Institute)<br>RIVM (National Institute for Public<br>Health and the Environment)
funding	lack of funding to improve FH care and/or informing families/awareness

Supplementary Material S1: Interview Protocol Stakeholder Views

Interview protocol, version April 2017

<u>Stakeholder analysis of the pros and cons of informing healthy individuals on their genome:</u>

Introduction:

-Introduce Members of the team

-Refer to the email and give the information letter.

-Give the consent form to sign and ask for consent to tape the interview.

Background (as discussed in the information letter)

In recent years, discussion has arisen how to effectively and responsibly use genomic information to prevent chronic disorders such as cancers and cardiovascular diseases. Important in this development is the possibility to offer preventive treatment to family members of identified index patients, such as in case of hereditary cancers and Familial Hypercholesterolemia. In European countries various strategies have been used to approach family members. A project funded by the European Commission (PRECeDI) allows us to conduct an interview study on the

question how actively family members could or should be approached and informed on their genetic risk. To study this question we contact stakeholders in FH care to discuss their views, and the pros and cons of current and alternative approaches.

Background to the questions:

In the Netherlands until 2013 an official screening programme for Familial Hypercholesterolemia existed to actively identify index patients. After patients were diagnosed in a very pro-active way their family members were contacted. StOEH invited family members after patient consent, who were visited at home and entire family groups were subsequently informed and tested together. Since the Netherlands has abandoned this approach the number of tested individuals has dropped from several thousands to several hundreds per year.

-What is your current role in FH care or organisation?

We would like to learn more about your views regarding proactive informing and contacting of family members of FH patients as in the time of the screening programme.

-Could you reflect on what would be the pros and cons of a pro-active approach of informing family members?

(Consider from literature: paternalism, forcing to test, cost, organisation, versus uptake, duty to care)

-Do you think the nature of the disorder call for this type of approach?

(Consider from literature: availability of treatment options)

We would like to learn more about your views regarding pro-active informing and contacting of family members of FH patients in the current situation?

How pro-active would you like to be?

-How would you balance the pros and cons as you see them in your specific practice?

-What would be your main concerns in this process?

-What would you consider to be desirable to optimise your current practice?

-What stakeholders do you see or work with in optimising current practices?

(Check views on policy making, collaboration between disciplines, implementation of new technologies)

-What factors can you identify that help or hinder improving current practices in collaborating with these stakeholders?

(Check for **cultural factors** such as norms, views on genetic testing and counselling; and **structural factors** such as funding, referral routines
